# Bio-jETI: a framework for semantics-based service composition

**DOI:** 10.1186/1471-2105-10-S10-S8

**Published:** 2009-10-01

**Authors:** Anna-Lena Lamprecht, Tiziana Margaria, Bernhard Steffen

**Affiliations:** 1 Chair for Programming Systems, Dortmund University of Technology, Dortmund, D-44227 Germany; 2 Chair for Service and Software Engineering, Potsdam University, Potsdam, D-14882 Germany

**Keywords:** Model Check, Resource Description Framework, Service Composition, Synthesis Algorithm, Computation Tree Logic

## Abstract

**Background:**

The development of bioinformatics databases, algorithms, and tools throughout the last years has lead to a highly distributed world of bioinformatics services. Without adequate management and development support, *in silico* researchers are hardly able to exploit the potential of building complex, specialized analysis processes from these services. The Semantic Web aims at thoroughly equipping individual data and services with machine-processable meta-information, while workflow systems support the construction of service compositions. However, even in this combination, *in silico* researchers currently would have to deal manually with the service interfaces, the adequacy of the semantic annotations, type incompatibilities, and the consistency of service compositions.

**Results:**

In this paper, we demonstrate by means of two examples how Semantic Web technology together with an adequate domain modelling frees *in silico* researchers from dealing with interfaces, types, and inconsistencies. In Bio-jETI, bioinformatics services can be graphically combined to complex services without worrying about details of their interfaces or about type mismatches of the composition. These issues are taken care of at the semantic level by Bio-jETI's model checking and synthesis features. Whenever possible, they automatically resolve type mismatches in the considered service setting. Otherwise, they graphically indicate impossible/incorrect service combinations. In the latter case, the workflow developer may either modify his service composition using semantically similar services, or ask for help in developing the missing mediator that correctly bridges the detected type gap. Newly developed mediators should then be adequately annotated semantically, and added to the service library for later reuse in similar situations.

**Conclusion:**

We show the power of semantic annotations in an adequately modelled and semantically enabled domain setting. Using model checking and synthesis methods, users may orchestrate complex processes from a wealth of heterogeneous services without worrying about interfaces and (type) consistency. The success of this method strongly depends on a careful semantic annotation of the provided services and on its consequent exploitation for analysis, validation, and synthesis. We are convinced that these annotations will become standard, as they will become preconditions for the success and widespread use of (preferred) services in the Semantic Web.

## Background

Research projects in modern molecular biology rely on increasingly complex combinations of computational methods to handle the data that is produced in the life science laboratories. A variety of bioinformatics databases, algorithms and tools is available for specific analysis tasks. Their combination to solve a specific biological question defines more or less complex analysis *workflows* or *processes*. Software systems that facilitate their systematic development and automation [1–7] have found a great popularity in the community.

More than in other domains the heterogeneous services world in bioinformatics demands for a methodology to classify and relate resources in a both human and machine accessible manner. The Semantic Web [8, 9], which is meant to address exactly this challenge, is currently one of the most ambitious projects in computer science. Collective efforts have already lead to a basis of standards for semantic service descriptions and meta-information.

Most importantly, the World Wide Web Consortium (W3C) set up a number of working groups addressing different technological aspects of the Semantic Web vision. Among their outcomes are the Semantic Annotations for WSDL (SAWSDL) recommendation [10], the Resource Description Framework (RDF) specification [11], and the Web Ontology Language (OWL) [12]. While SAWSDL is designed to equip single entities with predicates, RDF and the more powerful OWL formally define relationships between the resources of a domain.

Without a reasonably large set of semantically annotated (web) services, it is, however, difficult to evaluate the Semantic Web technologies with significant results and develop practical software for the client side. On the other hand, providers are not willing to put effort in annotating their services as long as they can not be confident which technologies will finally become established. Community initiatives like the Semantic Web Services (SWS) Challenge [13] or the Semantic Service Selection Contest (S3C) [14] address this problem. They provide collections of services, domain information and concrete scenarios that the different participants, being developers of methodologies for different Semantic Web aspects, have to deal with. In the scope of the S3 Contest, OPOSSum [15, 16], an "online portal to collect and share SWS descriptions" [16], was set up. It aims at collecting, sharing, editing, and comparing SWS descriptions within a community infrastructure in order to collaboratively evaluate and improve SWS formalisms. As of March 2009, however, OPOSSum does not list any bioinformatics services.

An example of a knowledge base particularly capturing bioinformatics data types and services are the constantly evolving namespace, object and service ontologies of the BioMoby service registry [17, 18]. BioMoby's aim is to "achieve a shared syntax, shared semantic, and discovery infrastructure suitable for bioinformat-ics" [19] as a part of the Semantic Web. Originating from the early 2000s, the 1.0 MOBY-S(ervices) spec-ifications, however, do not adhere to the Semantic Web standards that have been developed in the last years. Consequently, the S(emantic)-MOBY branch of the project came into being to migrate to common technologies. It has recently been merged into the SSWAP (Simple Semantic Web Architecture and Protocol) [20, 21] project, which aims at providing life science knowledge using standard RDF/OWL technology. SSWAP provides a number of own ontologies, but also incorporates third-party domain knowledge like the MOBY-S object and service ontologies.

Generally, the development of ontologies in the bioinformatics community is already very promising. Projects like the Gene Ontology (GO) [22] and the Open Biomedical Ontologies (OBO) [23] have already become widely used and also, for instance, incorporated by the SSWAP project. The majority of publicly available ontologies in the bioinformatics domain is, however, designed for the classification of scientific terms and the description of actual data sets, and not for (technical) descriptions of service interfaces and data types.

The lack of properly semantically annotated services has evidently already been recognized by the community, as different projects are commencing to address the issue. For instance, major service providers like the European Bioinformatics Institute (EBI) plan to extend their service infrastructure to provide meta-information conforming to Semantic Web standards. Other initiatives aim at setting up stand-alone collections of service URIs and corresponding annotations, without influencing the service infrastructures as such.

While the provision of semantically annotated services is mainly the service providers' task, on the client side software is needed that fully utilizes the available semantic information in order to provide helpful tools to the *in silico* researcher. The challenge for user-side software is to abstract from the underlying Semantic Web technology again and provide the achievements in an intuitive fashion.

**Figure 1:**
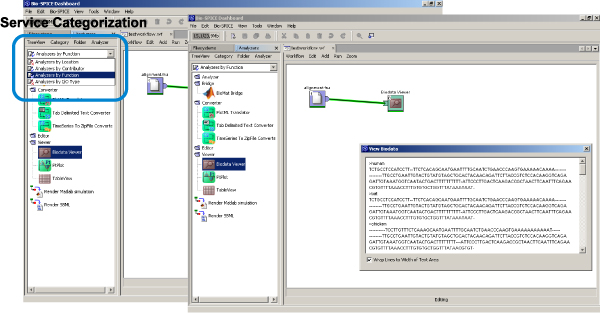
**BioSPICE Dashboard**. Graphical user interface of the BioSPICE Dashboard [24]. Services can be arranged according to the categories location, contributor, function, and I/O type (top left).

A simple but useful feature building upon semantic information about services is the categorization of services according to different criteria. A corresponding functionality has already been available in the meanwhile discontinued BioSPICE Dashboard, where it was possible to arrange services by location, provider, function, or I/O type (see Figure 1). The BioSPICE project is a meanwhile abandoned initiative that focused on the development of computational models for intracellular processes. Besides the provision of mere "access to the most current computational tools for biologists" [24], the work also aimed at integrating the services into a convenient graphical user environment, called the BioSPICE Dashboard. Thus, the need of multi-faceted service classification has been recognized several years ago, but until present services hardly provide appropriate meta-information.

More advanced examples of utilizing semantic information about services are, for instance, available in the scope of the SWS Challenge [13]. Among others, projects like SWE-ET (Semantic Web Engineering Environment and Tools) [25] and WSMX [26] participate in the challenge, adressing both discovery and mediation scenarios for Semantic Web Services. However, these solutions demand quite some technical understanding from the user, which hampers the uptake by a larger biological user community.

As an example from the bioinformatics domain, the BioMoby project provides a simple composition functionality for its services. [17, 18]. With the MOBY-S Web Service Browser [27] it is, e.g., possible to search for an appropriate next service, while in addition the sequence of actually executed tools is recorded and stored as a Taverna [4] workflow. A substantial drawback of this approach is, however, its restriction to the services that are registered in the respective platform.

In this paper, we present our approach to semantics-based service composition in the Bio-jETI platform [7, 28]. By integration of automatic service composition functionality into an intuitive, graphical process management framework, we are able to maintain the usability of the latter for semantically aware workflow development. Furthermore, we can integrate services and domain knowledge from any kind of heterogeneous resource at any location, and are not restricted to any semantically annotated services of a particular platform.

This manuscript is structured as follows: In the next section, Results and Discussion, we discuss two examples that we developed in Bio-jETI with the help of a semantics-aware workflow synthesis method and model checking: a simple phylogenetic analysis workflow and a more sophisticicated, highly customized phylogenetic analysis process based on Blast and ClustalW. Subsequently, the Conclusion deals with directives for the future development of our approach. Finally, the Methods section describes the applied techniques in greater detail.

## Results and discussion

**Figure 2:**
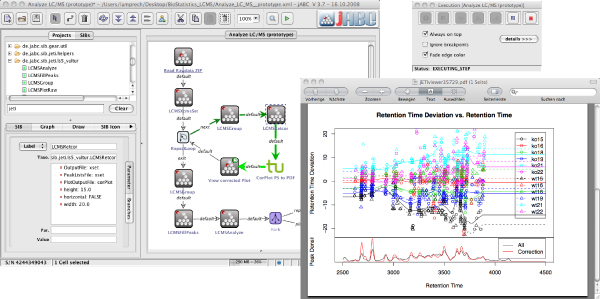
**Bio-jETI GUI**. The jABC framework, which provides the graphical user interface for Bio-jETI, supports the orchestration of processes from heterogeneous services. Workflow models are constructed graphically by placing process building blocks from a library (top left) on a canvas (center) and connecting them by labeled branches to define the flow of control. The models are directly executable by an inbuilt interpreter component (right).

**Figure 3:**
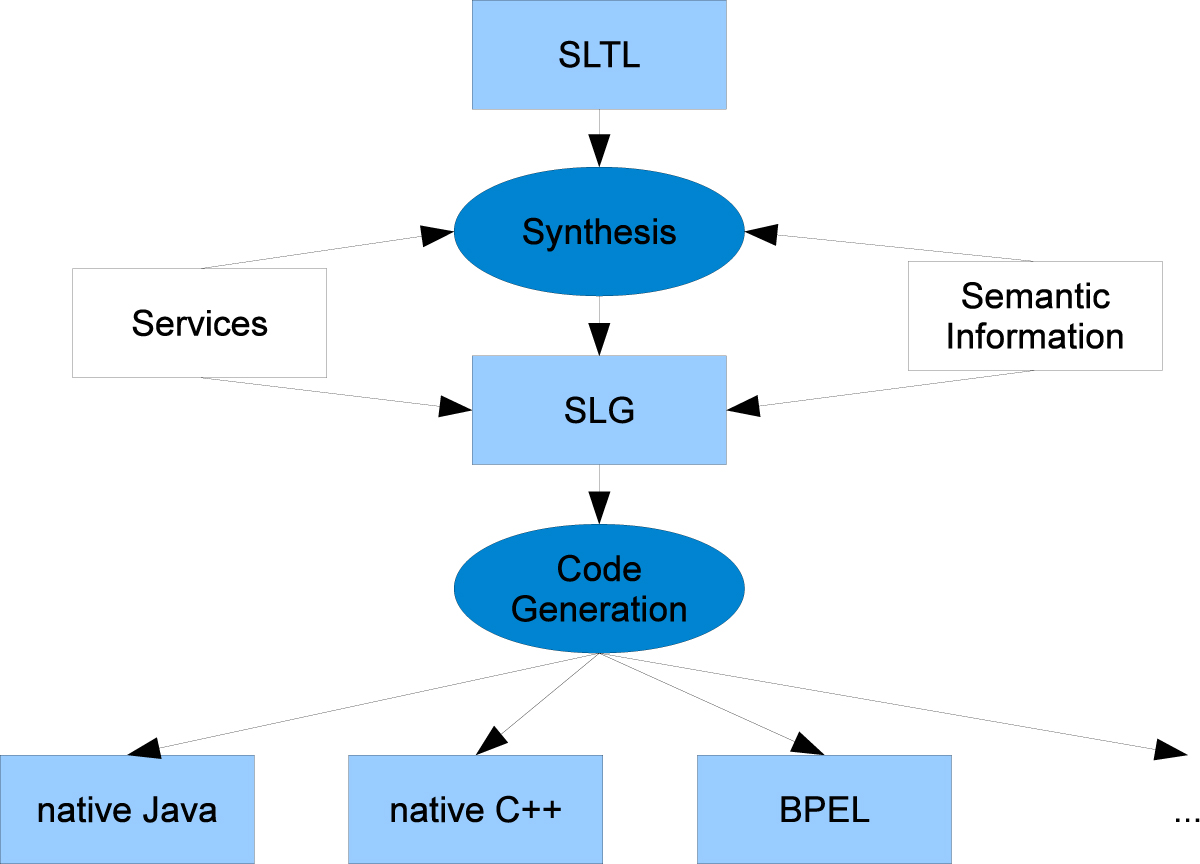
**Relationship between SLTL and workflow languages**. SLTL is designed to specify linear workflows on an abstract level. In conjunction with a set of services and adequate semantic information about the domain, it serves as input for the synthesis algorithm, which generates linear workflows according to the SLTL specification. The results are available as Bio-jETI SLGs, which can be further edited, combined, and refined. The SLGs can then be compiled into a number of different target languages by the GeneSys code generation framework.

The approach to semantics-based service composition that we present in this paper builds upon the Bio-jETI [7, 28] framework for model-based, graphical design, execution and management of bioinformatics analysis processes. It has been used in a number of different bioinformatics projects [29–32] and is continuously evolving as new service libraries and service and software technologies become established. Technically, Bio-jETI uses the jABC modeling framework [33, 34] as an intuitive, graphical user interface and the jETI electronic tool integration platform [35, 36] for dealing with remote services. Using the jABC technology, process models, called *Service Logic Graphs* (*SLGs*) are constructed graphically by placing process building blocks, called *Service Independent Building Blocks* (*SIBs*), on a canvas and connecting them according to the flow of control. Figure 2 shows a screenshot of the graphical user interface of the jABC. SLGs are directly executable by an interpreter component, and they can be compiled into a variety of target languages via the GeneSys code generation framework [37]. As Figure 3 (bottom) illustrates, GeneSys provides the means for transforming SLGs into native, stand-alone programm code (e.g., Java, C++) as well as into other workflow languages (e.g., BPEL).

Workflow development in Bio-jETI is already supported by several plugins of the jABC framework, for instance providing functionality for component validation or step-wise execution of the process model for debugging purposes. Now we are going to exploit further jABC technology, such as model checking and workflow synthesis, in order to enable Bio-jETI to support the development of processes in terms of service semantics.

Model checking [38, 39] can be used for reasoning about properties of process models. This can help to detect problems like undefined data identifiers, missing computations, or type mismatches. Solving these problems might require the introduction of further computational steps, for instance a series of conversion services in case of a data type mismatch. The approach here is to automate the creation of such process parts via workflow synthesis methodology [40–43] that allows for the automatic creation of (linear) workflows according to high-level, logical specifications. Figure 3 (top) illustrates the relationship between our specification language SLTL (Semantic Linear Time Logic) and the actual Bio-jETI workflow models, the SLGs: Provided with a logical specification of the process and semantically annotated services, the workflow synthesis algorithm generates linear sequences of services, which can be further edited and combined into complex process models on the SLG level.

For the study that we present in this paper we used a SIB collection offering various remote and local services. Examples for contained remote services are the data retrieval services provided by the EBI (European Bioinformatics Institute) [44, 45], sequence analysis algorithms offered by BiBiServ (the Bielefeld Bioinformatics Server) [46], web services hosted by the DDBJ (DNA Data Bank of Japan) [47], and some tools of the EMBOSS suite [48]. On the local side, there are specialized components such as visualizer for phylogenetic trees [49] and more generic ones like SIBs that realize user interaction or functionality for file management. Table 1 lists the fragment of the library that is relevant for our examples.

**Table 1:** Exemplary set of services. Fragment of a component library that we used in the examples. The table lists the names of the building blocks (SIBs) along with function descriptions and selected service predicates.

SIB	Description
Archaeopteryx	Displays a phylogenetic tree [49].
	type:visualization, location:local, contributor:forester.org
BLAST	BLAST [63] against a DDBJ database.
	type:analysis, location:ddbj, contributor:ddbj
ClustalW	Runs ClustalW [64].
	type:analysis, location:ddbj, contributor:ddbj
Emma	EMBOSS [48] interface to ClustalW.
	type:analysis, location:ebi, contributor:emboss
ExtractPattern	Extracts all parts of a string that match a regular expression.
	type:stringprocessing, location:local, contributor:jabc
GetDDBJEntry	Fetches an entry in at file format from a DDBJ database [51].
	type:dataretrieval, location:ddbj, contributor:ddbj
GetFASTA_DDBJEntry	Fetches an entry in FASTA format from a DDBJ database [51].
	type:dataretrieval, location:ddbj, contributor:ddbj
List2String	Concatenates all entries of a list.
	type:stringprocessing, location:local, contributor:jabc
MatchString	Tries to match a string against a regular expression pattern.
	type:condition, location:local, contributor:jabc
PutExpression	Stores a user-supplied context expression or its value into the execution context.
	type:definition, location:local, contributor:jabc
PutInteger	Provides an integer value.
	type:definition, location:local, contributor:jabc
RepeatLoop	Realizes a counting loop.
	type:loop, location:local, contributor:jabc
ReplaceString	Replaces substrings of a string with another character sequence.
	type:stringprocessing, location:local, contributor:jabc
ShowInputDialog	Input dialog, provides a string.
	type:definition, location:local, contributor:jabc
WSDBFetch	Gets sequences from an EBI database [44, 45].
	type:dataretrieval, location:ebi, contributor:ebi

In the jABC, the SIBs are displayed to the user in a taxonomic view, classified according to their position in the file system (by default) or to any other useful criterion, like the provider or the kind of service. The SIBs have user-level documentation, explaining what the underlying tool or algorithm does, that is derived directly from the provider's service descriptions. In addition, the SIBs provide information about their input and output types via a specific interface. This is already an integral part of the semantic information that helps to systematically survey large SIB libraries and it is used by our process synthesis and model checking methods. It is, in addition, possible to add arbitrary annotations to the SIB instances and by doing so providing further (semantic) information that is taken into account by our formal methodologies.

The knowledge base that is needed for the process synthesis consists, furthermore, of service and type taxonomies that classify the services and types, respectively. Taxonomies are simple ontologies that relate entities in terms of *is-a* and *has-a* relations. These classifications provide sufficient information for our synthesis methodologies.

**Figure 4:**
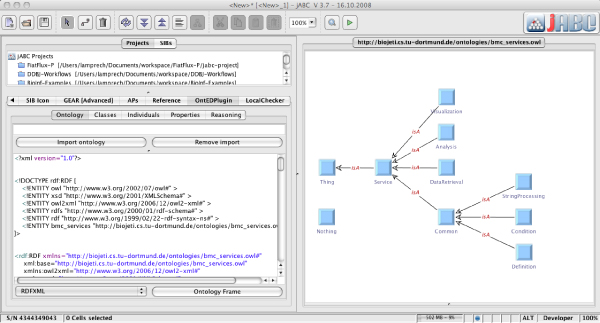
**Service taxonomy**. Service taxonomy for the services that we use in our examples, edited in OntEd, the ontology editing plugin of the jABC.

**Figure 5:**
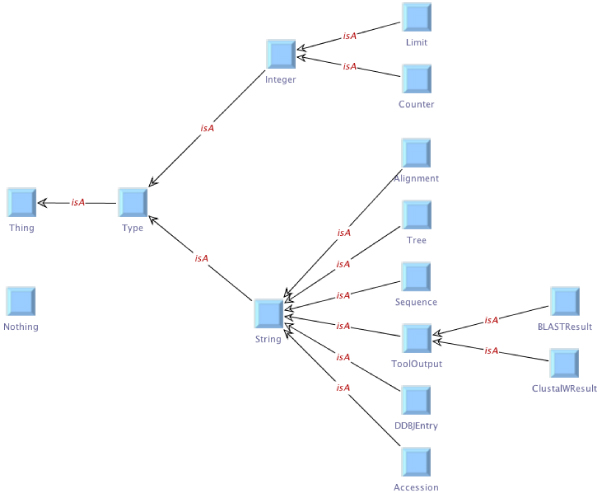
**Type taxonomy**. Type taxonomy classifying the data types involved in our examples.

We assume simple taxonomies for our examples, which have the generic OWL type Thing at the root. Going downwards, classifications are introduced, for instance refining the generic type into integers and strings, whereas the latter is further distinguished into alignments, trees, sequences, tool outputs, and so on. Figure 4 shows the service taxonomy for the services that we use in our examples, edited in the OntEd ontology editor plugin of the jABC. The corresponding type taxonomy classifying the involved data types is given in Figure 5.

The basic input and output information for the services is defined in terms of the data types contained in the type taxonomy. Table 2 lists the set of data types that is relevant for our examples. The services are characterized by input-output-pairs of types, where the input or output may well be empty (as it is the case, e.g., for ShowInputDialog and Archaeopteryx), respectively. Services may also provide multiple possible transformations and thus achieve polymorphism. For instance, BiBiServ's ClustalW can process sequences in FASTA or in SequenceML format, and produces a FASTA or AlignmentML output, accordingly.

**Table 2:** Exemplary set of types. The set of data types that was used in the example processes.

Type	Description
Accession	Single accession number.
AccessionList	Iteratable (java.util.)list of accession numbers.
Accessions	Concatenation of accession numbers, separated by some character.
Alignment	Multiple sequence alignment.
BlastResult	Tool output of BLAST.
ClustalWResult	Tool output of ClustalW.
Counter	Counter, i.e. positive integer value.
DDBJEntry	DDBJ entry in flat file format.
Limit	Limit, i.e. positive integer value.
Sequence	Single or multiple nucleic or amino acid sequences.
Tree	Phylogenetic tree.

### Example 1: a simple phylogenetic analysis workflow

When developing bioinformatics analysis workflows, users often have a clear idea about the inputs and final results, while their conception of the process that actually produces the desired outputs is only vague. Figure 5 (upper left) shows a stub for a workflow: the start SIB (left) is an input dialog for a nucleic or amino acid sequence, which is followed by a SIB running a BLAST query with the sequence having been input in order to find homologous sequences. The workflow ends by invoking Archaeopteryx to display a phylogenetic tree (right). The configuration of the SIBs is sound at the component level, as the Local Checker plugin (producing the small overlay icons top left) confirms. However, there are errors regarding the correct configuration of the model as a whole, as the required input type for Archaeopteryx, some phylogenetic tree format, is not produced previously in the process. This is detected by our model checker GEAR (indicated by a red overlay icon with a white cross in the top right corner of the SIBs), that checks a temporal formula covering the following constraint (please refer to the Methods section for details on the model checking procedure):

An experienced bioinformatician might be aware of the problem immediately, due to his familiarity with the involved tools. This is, however, only a small workflow. An automatic, semantically supported detection of misconfigurations and modeling errors unfolds its full potential when processes become more complex, and it is not feasible for the *in silico* researcher to dive into the documentations of all services or to explore their behaviour by trial-and-error executions.

Once detected, there are different ways to fix the problem. One can look for replacements for one of the involved SIBs that essentially compute the same results, but provide them in a data format that fits in the surrounding process. Another approach, assuming that the user has chosen these services for good reason, is to search for a sequence of additional services that resolve the mismatch and insert them into the process. Such data mediation sub-workflows are usually linear. They can consist of type conversions that simply adapt the involved data, or also of real computational services when the match can not be realized so easily.

**Figure 6:**
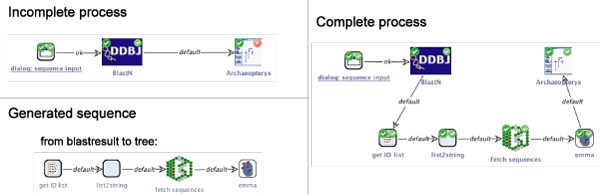
**Example 1**. A simple phylogenetic analysis process. The upper left shows an erroneous stub for a simple phylogenetic analysis process, it lacks a sequence of services leading from a BLAST result to a phylogenetic tree. Below is the appropriate sequence of services that is proposed by our synthesis algorithm. The complete and correct analysis process is shown on the right.

As a means for resolving the violation of property *, the example process model stub implies a process specification adequate as input for our workflow synthesis algorithm (please refer to the Methods section for details). In a high-level formulation, it reads:

Utilizing the semantically annotated SIB collection and domain information from above, and computing the shortest service combination that satisfies the specification, our synthesis algorithm proposes the following simple four-step workflow for the above query (bottom left in Figure 6):

1. Extract the IDs of the hits from the BLAST result (using a regular expression).
2. Turn the matches into a comma-separated list.
3. Call DBFetch (fetching the corresponding sequences from a database).
4. Run emma (computing a multiple sequence alignment and phylogenetic tree).

**Figure 7:**
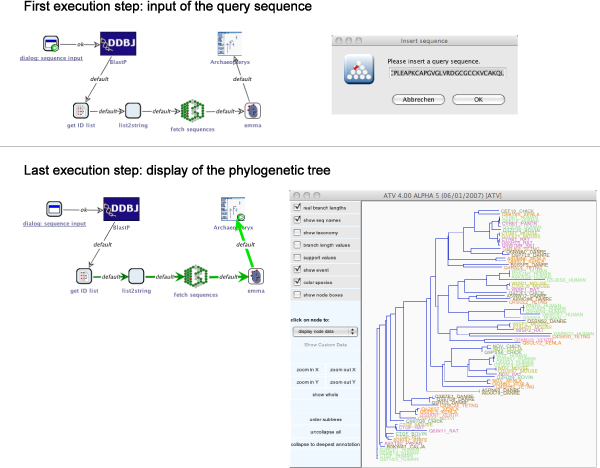
**Execution of example 1**. Execution of the simple phylogenetic analysis process. The execution begins with an interactive step, where a dialog is displayed in which the query sequence is entered (top). After some non-interactive steps, the finally available phylogenetic tree is displayed using Archaeopteryx (bottom).

The generated sequence of SIBs can now be inserted into the process stub and all parameters configured appropriately. As Figure 6 (right) shows, neither the local nor the model checking does reveal errors any more. The process is now ready for execution. Figure 7 illustrates the corresponding runtime behaviour: the workflow starts by asking the user for a query sequence, then performs a similarity search, data retrieval and sequence analysis before it finally displays the resulting phylogenetic tree.

### Example 2: Blast-ClustalW workflow

A simple phylogenetic analysis like in the previous example is an often recurring element of complex *in silico* experiments. In many cases, however, a customized, more specific processing of intermediate results is required, like in the Blast-ClustalW workflow [50] that is one of the DDBJ's sample workflows for the Web API for bioinformatics [51]. It is the archetype for our second example.

**Figure 8:**
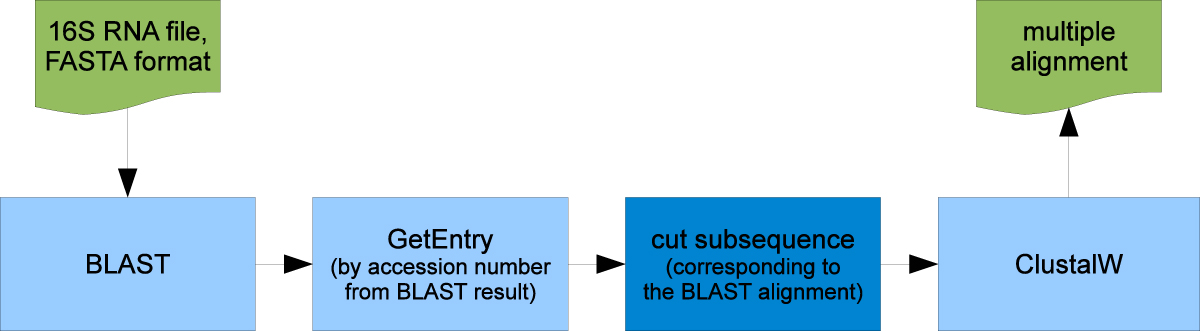
**Blast-ClustalW workflow**. Blast-ClustalW workflow as sketched by the DDBJ (following [50]).

The Blast-ClustalW workflow [50] has the same inputs and outputs as the simple phylogenetic workflow from example 1: It finds homologuous sequences for an input DNA sequence via BLAST and computes a hypothesis about the phylogenetic relationship of the obtained sequences (using ClustalW). The proposed analysis procedure consists of four major computation steps (the blue rectangles in Figure 8, whereby steps 2 and 3 have to be repeated for each Blast hit that is taken into account (not evident from the figure):

1. Call the Blast web service to search the DDBJ database for homologues of a nucleic acid sequence. The input is a 16S RNA sequence in FASTA format, the output lists the database IDs of the similar sequences and basic information about the local alignment, e.g. its range within the sequences.
2. Call the GetEntry web service with a database ID from the Blast output to retrieve the corresponding database entry.
3. Extract accession number, organism name and sequence from the database entry. Trim the sequence to the relevant region using the start and end positions of the local alignment that are available from the BLAST result.
4. Call the ClustalW web service to compute a global alignment and a phylogenetic tree for the prepared sequences.

Due to the loop that is required for repeating steps 2 and 3 a certain number of times, this process can not be created completely by our current synthesis algorithm, which is restricted to produce linear sequences of services. It is, however, possible to predefine a sparse process model in which the looping behaviour and other crucial parts are manually predefined, and to subsequently fill in linear parts of the process automatically.

**Figure 9:**
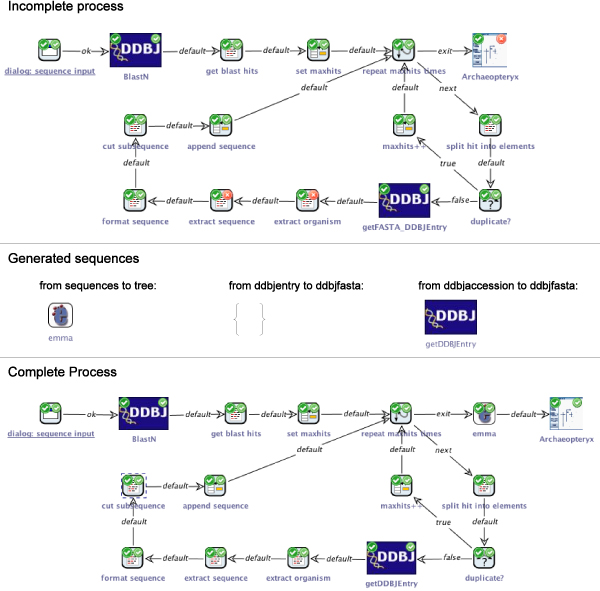
**Example 2**. The more complex Blast-ClustalW workflow. The model checking detects three errors for the original process (top). To bridge the gap between the available sequences and the required tree, the emma web service can be inserted, computing a multiple alignment and providing the corresponding phylogenetic tree. No mediating sequence can be found that converts DDBJ entry into FASTA format, but it is possible to get this format when the also available DDBJ accession number is used as input (center). The complete process (bottom) has an additional SIB emma and has substituted getFASTA_DDBJEntry by getDDBJEntry.

Figure 9 (top) shows an advanced, but still incomplete model of the Blast-ClustalW workflow. Like in example 1, the process begins with displaying a dialog for entering the query sequence (start SIB top left). The result of the subsequent Blast web service invocation is split into the separate results (SIB get blast hits). Before the loop is entered, a maximum is set for number of hits that is to be considered in the analysis. For this defined maximum number of hits, the loop's body is executed. The current hit is split into its seperate elements, e.g. accession number, score, and the start and end position of the local alignment that produced by BLAST within the whole sequence. The accession number is used to check whether the sequence corresponding to the current hit has already been added to the analysis in order to avoid duplicate sequences. If a duplicate is detected, the maximimum number of hits is incremented, so that another hit can be taken into account. Otherwise, the corresponding entry is fetched from the database using the DDBJ's GetEntry web service (SIB getFASTA_DDBJEntry). The SIBs extract organism and extract sequence are then applied to extract the corresponding information from the DDBJ entry by means of a regular expression. The sequence is formatted, i.e. whitespaces removed, and the start and end positions that are known from the BLAST result are used to cut the subsequence that actually contributed to the local alignment during the BLAST search. The prepared sequence is then added to the analysis (SIB append sequence). Note that in contrast to the original representation of Figure 8, we see here the structure and the data-driven loops of the actual workflow. Finally, the resulting phylogenetic tree is displayed by Archaeopteryx.

At this state of the process, the local checking of the components detects no errors, but the model checker reveals problems (overlay icons top right): As in the previous example, the SIB Archaeopteryx uses a variable tree, which is not defined before. Moreover, the SIBs extract organism and extract sequence use a variable ddbjentry, which is defined with an incompatible type. Details on the model checking procedure can be found in the Methods section.

To resolve the first problem, we proceed similar as in example 1, by providing the synthesis algorithm with a temporal formula that asks for a sequence of services that takes a set of sequences as input (which is the last intermediate result that is computed previous to Archaeopteryx in the process) and produces a phylogenetic tree (the input that Archaeopteryx expects). As Figure 9 (center) shows, a single call to emma is one of the (shortest) sequences that fulfils this request.

The second problem is the presence of a type ddbjfasta where the type ddbjentry is expected. To solve this mismatch, we ask our synthesis algorithm for a way to derive the latter from the former. It returns with an empty result (see Figure 9, center), which means that our SIB collection can not provide an appropriate sequence of services. We exclude the type ddbjfasta and the SIB getFASTA_DDBJEntry, by which is it produced, and try our luck with the type ddbjaccession, which has been defined last, as starting point for the synthesis. The answer is a service sequence consisting of the SIB getDDBJEntry (center), by which we can now substitute the improper data retrieval SIB from above.

The bottom of Figure 9 shows the completely assembled process. We omit to demonstrate its execution behaviour, as it is very similar to that of example 1.

### Discussion and perspectives

By means of two examples, the previous sections demonstrated the local checking, model checking and workflow synthesis methodology that is currently available in the jABC framework and thus part of Bio-jETI. The Local Checker plugin provides domain-independent functionality and is already conveniently integrated in the framework. We are now working on a user-friendly integration of the domain-specific model checking and synthesis techniques, especially with regard to the bioinformatics application domain. This ongoing work spans three dimensions, which are discussed in the following sections: domain modeling, model checking, and model synthesis.

#### Domain modeling

This dimension is the heart of making information technology available to biologists, as it enables them to express their problems in their own language terms – on the basis of adequately designed ontologies. It raises the issue where the domain knowledge ideally comes from. It is, of course, possible for each user to define custom service and type taxonomies, allowing for exactly the generalization and refinement that is required for the special case. However, as the tools and algorithms that are used are mostly third-party services, it is desirable to automatically retrieve domain information from a public knowledge repository as well. Therefore we plan to incorporate knowledge from different publicly available ontologies, like BioMoby [17, 18] and SSWAP [20, 21], and to integrate it into the service and type taxonomies for use by our synthesis methodology.

It is, of course, also necessary that the services themselves are equipped with meta-information in terms of these ontologies. Again, we are looking at BioMoby with interest: numerous institutions have registered their web services at Moby Central, describing functionality and data types in pre-defined structures using a common terminology. Although BioMoby does not yet use standardized description formalisms like SAWSDL, it is already clear that there is semantic information available that we can use as predicates for automatic service classification.

Furthermore it will be interesting to consider the incorporation of more content-oriented ontologies like the Gene Ontology [22] or the OBO (Open Biomedical Ontologies) [23] into our process development framework. This would allow the software to not only support the process development on a technical level, but also in terms of the underlying biological and experimental questions. Additional sources of information, like the provenance ontologies of [52] could be also easily exploited by our synthesis and verification methods.

#### Model checking

This dimension is meant to systematically and automatically provide biologists with the required IT knowledge in a seamless way, similar to a spell checker which hints at orthographical mistakes – perhaps already indicating a proposal for correction. Immediate concrete examples of detectable issues are (cf. the examples presented earlier):

- Missing resources: a process step is missing, so that a required resource is not fetched/produced.
- Mismatching data types: a certain service is not able to work on the data format provided by its predecessor.

However, this is only a first step. Based on adequate domain modeling, made explicit via ontologies/taxonomies, model checking can capture semantic properties to guarantee not only the executability of the biological analysis process but also a good deal of its purpose, and rules of best practice, like:

- All experimental data will eventually be stored in the project repository.
- Unexpected analysis results will always lead to an alert.
- Chargeable services will not be called before permission is given by the user.

On a more technical side, model checking allows us also to apply the mature process analysis methodology that has been established in programming language compilers in the last decades [53] and has shown to be realizable via model checking [54, 55]. By providing a predefined set of desirable process properties to the model checker we plan to achieve a thorough monitoring of safety and liveness properties within the framework. Similar to the built-in code checks that most Integrated (Software) Development Environments provide, this would help Bio-jETI users to avoid the most common mistakes at process design time. In addition, the list of verified properties is extendable by the user, and can thus be easily adapted to specific requirements of the application domain.

#### Model synthesis

This dimension can be seen as a step beyond model checking: The biologist does not have to care about data types at all – the synthesis automatically makes the match by inserting required transformation programs. This is similar to a spell checker which automatically corrects the text, thus freeing the writer from dealing with orthography at all. (In our model-based framework, things are well-founded, without the uncertainties of natural language. Please do not be put off by this example because of annoying experiences with spell checkers!)

The potential of this technology goes even further: ultimately, biologists will be able to specify their requests in a very sparse way, e.g. by just giving the essential corner stones, and the synthesis will complete this request to a running process. In our text writing analogy, this might look like a mechanism that automatically generates syntactically and intentionally correct text from text fragments according to predefined rules that capture syntax and intention. For instance, the fragments "ten cars", "1000 Euro for shipping", "19% value added tax", "four days" and "Mercedes", may be sufficient to synthesize a letter in which a logistics company offers its services to Mercedes according to a specific request.

Back to biology, the fragments "DNA sequences", "phylogenetic tree", and "visualization", may automatically lead to a process that fetches EBI sequence data, sends them in adequate form to a tool that is able to produce a phylogenetic tree, and then transfers the result to an adequate viewer. Typically there are many processes that solve such a request. Thus our synthesis algorithm provides the choice of producing a default solution according to a predefined heuristics, or to propose sets of alternative solutions for the biologist to select.

## Conclusion

We demonstrated by means of two examples how Semantic Web technology together with an adequate domain modelling frees *in silico* researchers from dealing with interfaces, types, and inconsistencies. In Bio-jETI, bioinformatics services can be graphically combined to complex services without worrying about details of their interfaces or about type mismatches of the composition. These issues are taken care of at the semantic level by Bio-jETI's model checking and synthesis features. Whenever possible, they automatically resolve type mismatches in the considered service setting. Otherwise, they graphically indicate impossible/incorrect service combinations. In the latter case, the workflow developer may either modify his service composition using semantically similar services, or ask for help in developing the missing mediator that correctly bridges the detected type gap. Newly developed mediators should then be adequately annotated semantically, and added to the service library for later reuse in similar situations.

In the first example we developed a simple phylogenetic analysis workflow. The model checker detected a SIB trying to access a data item that has not been defined previously in the workflow, which indicates that necessary computation steps are missing. We used the synthesis algorithm to generate the sequence of these missing steps.

The second example dealt with a more complex phylogenetic analysis workflow, involving several local steps processing intermediate data. Here, the model checker did not only detect missing computations, but also a type mismatch that lead to an incorrect process model. Again, the synthesis algorithm was used to find an appropriate intermediate sequence of services and an alternative to the erroneous part of the workflow, respectively.

We believe that our model checking and synthesis technologies have great potential with respect to making highly heterogeneous services accessible to *in silico* researchers that need to design and manage complex bioinformatics analysis processes. Our approach aims at lowering the required technical knowledge according to the *"easy for the many, difficult for the few"* paradigm [56]. After an adequate domain modeling, including the definition of the semantic rules to be checked by the model checker or to be exploited during model synthesis, biologists should ultimately be able to profitably and efficiently work with a world-wide distributed collection of tools and data, using their own domain language. This goal differentiates us from other workflow development frameworks like Kepler [3] or Triana [5], which can be seen as middleware systems that facilitate the development of grid applications in a workflow-oriented fashion. They require quite some technical knowledge. In Kepler, for instance, the workflow design involves choosing an appropriate *Director* for the execution, depending on, e.g., whether the workflow depends on time, requires multiple threads or distributed execution, or performs simple transformations. These aspects have to be taken into account for efficient execution of complex computiations, but not necessarily when dealing with the actual composition of services. This way, these frameworks address a bioinformatics user, and not the biologists themselves.

We believe that Bio-jETI's control flow-oriented approach is suitable for adressing non-IT personnel: it allows them to continue to think in "Dos" and "Dont's", and steps and sequences of action in their own terms at their level of domain knowledge. In contrast, dataflow-oriented tools like Kepler [3], Taverna [4], or Triana [5] require their users to change the perspective to a resource point of view, which, in fact, requires implicit (technical) knowlegde to profitably use them.

The challenge for us is now to integrate the available semantic information and the semantically aware technologies into our process development framework in the most user-convenient way. One central issue is to find an appropriate level of abstraction from the underlying technology: we would like to provide a set of general, pre-defined analyses and synthesis patterns, but at the same time give experienced users a way to add specialized specifications. Another issue is how to integrate semantic information about the application domain and its services into this (partly) automated workflow development process, since such knowledge is essential to achieve adequate results.

On the one hand, this requires predicates characterizing the single services, i.e. their function and their input/output behaviour. On the other hand, taxonomies or ontologies are required which provide the domain knowledge against which the services (their predicates) are classified. The majority of this information has to be delivered by the tool and database providers, covering semantics of services as well as semantics of data. The convenience on the client side will increase as the Semantic Web spreads and new standards become established.

## Methods

This section describes the methodologies for process model verification and synthesis that we used for developing the presented examples.

### Process model verification via model checking

Model checking provides a powerful mechanism to analyze and verify static aspects of (arbitrary) models. Generally speaking, it can be used to check whether a model *M* satisfies a property *ϕ*, usually written as

where *ϕ* is expressed in terms of a modal or temporal logic. Applying model checking to process models can help to detect problems in the design phase. It is in particular useful to analyze aspects of the whole model, where syntax or type checking at the component level is not sufficient. Examples for errors whose detection requires awareness of the whole model are manifold, ranging from undefined variables or simple type mismatches to computational gaps and incomplete processes. The list of properties against which the model is evaluated is easily extendable, since including a new constraint in the verification only requires to write a modal or temporal formula expressing the property of interest.

The model checker GEAR [39] allows to evaluate static properties of models within the jABC, basically using the *Computation Tree Logic* (CTL) [57] to formulate appropriate constraints. CTL is a temporal, branching time logic designed to reason about models represented as directed graphs, and whose syntax can be described by the following BNF:

Thus, in addition to the operations and operands known from propositional logic, it comprises the modalities *AF*, *EF*, *AG*, *EG*, *AU*, and *EU*. The *A* s and *E* s are *path-quantifiers*, providing a universal (*A*) or existential (*E*) quantification over the paths beginning at a state. *F*, *G*, and *U* express *linear-time modalities* for the path, specifying that *ϕ* must hold finally (*F*), generally (*G*), or that *ϕ*_1_ has to be valid until *ϕ*_2_ finally holds (*U*). For example, the CTL formula

expresses that on all paths through the model that begin at the considered state, finally Rome is reached. As another example from routing, the formula

states that there is a path that is completely (globally) free of charge. That all routes should be toll-free until a particular place, say Rome, is reached, can expressed using the *U* ntil operator:

GEAR extends this variant of CTL further and includes additional overlined modalities representing a backward view, i.e. considering the paths that end at a given state. We apply it to our (bioinformatics) process models, the Service Logic Graphs (SLGs), where the entire processes are the models, the individual activities (the services, in the form of SIBs) are the nodes, and the edges express the conditional flow of control. As both nodes and edges are labeled, these models are formally so-called Kripke Transition Systems.

**Figure 10:**
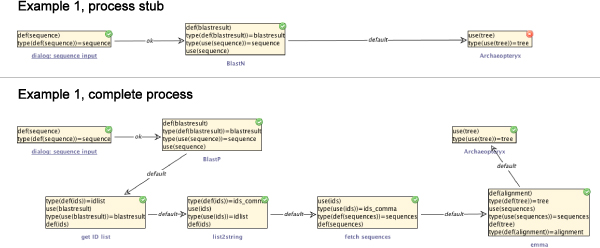
**Atomic propositions of the SIBs in example 1**. Atomic propositions of the process stub and complete process model of example 1. The propositions describe basic data flow properties, like defined and used variables and their types in terms of the data types of the taxonomy.

Basis for the analysis of processes are the *atomic propositions*, simple properties that hold for single nodes of the process model. For instance, we can add an atomic proposition use(x) to a SIB to express that a data item x is used by the service, or def(x) to state that it is produced (defined). Furthermore we can assume to have information about the types of the input and output data, and denote that a used or defined item x is of type y by type(use(x)) = y and type(def(x)) = y, respectively. Figure 10 shows the atomic propositions of the SIBs in example 1.

As we have seen in the examples, a model property of interest for processes orchestrated from remote services could be

The dependence between the two parts of this requirement is a usual Boolean implication, the temporal constraint in the second part is expressed by the backward CTL modality :

While this is sufficient to ensure that the variable x has been defined at all, it does not say anything about type correctness. Since the name x, however, could be used to refer to different data throughout the process, it is reasonable to extend the above constraint and to include the type of the used variable. In example 1, we considered, for instance, a variable of tree of type Tree:

*If a service uses a data item* tree *of type* tree, tree *must have been defined before with precisely this type, without having been overwritten since*.

The corresponding CTL formula is:

The model checking reveals a property violation, as can be seen in Figures 6 (top left) and 10 (top): the rightmost SIB is marked by a red overlay icon in the upper right corner, indicating that the property is violated at that node. The reason is that the process does not provide the appropriate input type for the tree visualizer. The same formula can be applied analogously to other variables with other types, as we did, for instance, in our second example.

### Process synthesis

By *process synthesis* we refer to techniques that construct workflows from sets of services according to logical specifications [58]. The algorithm that we use for our approach is based on a modal logic that combines relative time with descriptions and taxonomic classifications of types and services [40]. It was implemented for the ABC and ETI platforms [43, 59], and lately also used within the jABC framework. We applied it, for instance, in the SWS Challenge [13] to synthesize a mediator process converting between different message formats that were used by the web service providers in the scenario of [60, 61].

In the following we describe how to apply our synthesis method, i.e. 1) how the domain knowledge forms a configuration universe, 2) how a modal logic can be used for workflow specification, and 3) what the algorithm can finally derive from this information. Note that we focus on usage here, details on the underlying logics and algorithms can be found in [40, 59].

#### The configuration universe

**Figure 11:**
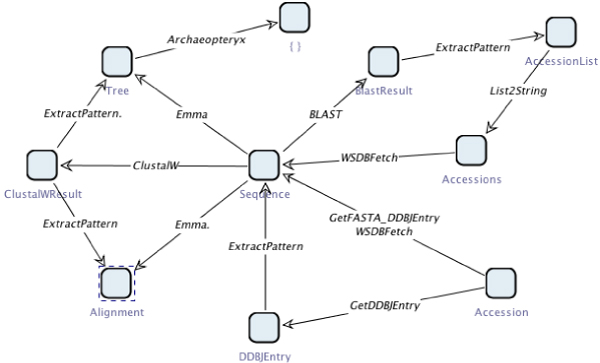
**Fragment of the configuration universe**. Fragment of the configuration universe based on the services and types from Tables 1 and 2. Paths through the configuration universe represent possible sequences of services. Note that the configuration universe is able to express service polymorphisms: the service ExtractPattern, for instance, can be applied to different inputs, and produces different outputs, accordingly.

The domain knowledge that has to be provided for our synthesis algorithm comprises basically three sets: types, services, and transitions. The set of types that is available in the domain form the static aspects, i.e. type constraints that are used as atomic propositions by the underlying logic. The set of services represents the dynamic aspects of the domain, which can be used as actions by the logic. According to the observation that tools and algorithms can simply be seen as transformations that take an input and produce a corresponding output [59], the set of transitions is given in triples of the form *(input, service, output)*. Together, types, services, and transitions form the configuration universe, in which each (finite) path represents a possible workflow. Figure 11 illustrates a configuration universe that is implied by the SIBs and data types of our examples. The synthesis algorithm searches the configuration universe for a path satisfying a particular specification.

In addition, the domain knowledge can be extended further by hierarchically organizing types and services in taxonomies, i.e. simple ontologies that relate entities in terms of *is-a* and *has-a* relations. The types and service taxonomies for our examples are given in Figures 4 and 5. The taxonomies are considered by the synthesis algorithm when evaluating type or service constraints.

#### The specification language

Our workflow specification language, which we call SLTL (for Semantic Linear Time Logic), can be seen as a linear-time variant of CTL (see previous section) or interpreted version of the Propositional Linear Time Logic. SLTL is described by the following BNF:

where *t*_*c*_and *s*_*c*_express type and service constraints, respectively.

Thus, SLTL combines static, dynamic, and temporal constraints. The static constraints are the taxonomic expressions (boolean connectives) over the types or classes of the type taxonomy. Analogously, the dynamic constraints are the taxonomic expressions over the services or classes of the service taxonomy. The temporal constraints are covered by the modal structure of the logic, suitable to express the order in which services can be combined.

A formal definition of the semantics of SLTL can be found in [40]. Intuitively, true is satisfied by every sequence of services, and *t*_*c*_by every sequence whose first component has an input interface satisfying *t*_*c*_. Negation and disjunction are interpreted in the usual fashion. The construct ⟨*s*_*c*_⟩*ϕ* is satisfied if the first component satisfies *s*_*c*_, and the continuation of the service sequence satsifies *ϕ*. A formula of the form *Gϕ* requires that *ϕ* is satisfied **G** enerally, and *ϕUψ* expresses that the property *ϕ* holds for all services of the sequence, **U** ntil a position is reached whare the corresponding continuation satisfies the property *ϕ*.

It is convenient to derive further operators from these basic constructs. The boolean disjunction

and the Eventually operator

are two common examples.

Coming to concrete examples of workflow specifications, the synthesis algorithm can be used to generate linear workflows just on the basis of an intial type (e.g. BlastResult) and a final type (e.g. Tree) via the following SLTL formula:

As we have seen in the workflow examples, already this simple query has a real practical impact, as it allows to autmatically resolve type mismachtes.

As another example, it is possible to query for an explicit sequence of services, let's say an input dialog asking for an accession number followed by the retrieval of the corresponding sequence from a database and a BLAST query:

Note that the service constraints in the formula are not concrete service names, but terms from the service taxonomy that define higher-order service categories. The synthesis algorithm takes care of instantiating the result with concrete services.

#### The synthesis algorithm

The synthesis algorithm interprets SLTL formulas over paths of the configuration universe, i.e. provided with a specification, it searches the configuration universe for (finite) corresponding paths. The algorithm is based on a tableau method, of which a detailed description is given in [40]. It automatically generates all, all minimal, or all shortest service compositions that satisfy a specification, according to the selected synthesis mode. The algorithm's output is the basis for the final assembly of the corresponding SLG.

**Figure 12:**
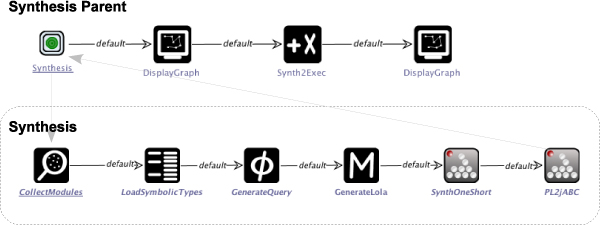
**Synthesis SLG**. Complete synthesis process, realized as jABC SLG. The main process (top) triggers the execution of the synthesis and displays the solution that is returned. Then, it assembles the SLG corresponding to this solution and displays it on a canvas, where it can be used for further process development. The actual synthesis is carried out by the sub-process (bottom): It captures the available domain knowledge and evaluates the workflow specification. The collected information is stored in a specific database file and sent to the synthesis algorithm, which computes one shortest solution (SynthOneShort). The generated sequence of services is then converted into the jABC's graph format in order to allow further processing within the framework.

The presently available implementation of the algorithm had been developed for use within the ABC, the jABC's predecessor that has been written in C++. In order to make it accessible from within the Java-based jABC framework, we integrated it using the jETI technology. The complete synthesis process is then defined by an SLG, as shown in Figure 12: The main process (top) triggers the execution of the synthesis and displays the solution that is returned. Then, it assembles the SLG corresponding to this solution and displays it on a canvas, where it can be used for further process development. The actual synthesis is carried out by the sub-process (bottom): It captures the available domain knowledge by collecting information about the available services (SIB CollectModules) and types (LoadSymbolicTypes) while evaluating the workflow specification (GenerateQuery). The collected information is stored in a specific database file (GenerateLola) and sent to the synthesis algorithm, which computes one shortes solution (SynthOneShort). The generated sequence of services is then converted into the jABC's graph format (PL2jABC) in order to allow further processing within the framework.

We are currently re-implemening the algorithm in Java, making it suitable for seamless integration into the jABC framework. Also, we will add functionality for facilitating the synthesis procedure for the user, for instance by providing a graphical interface supporting the domain modeling and formula patterns for the specification of workflows. Furthermore, we plan to incorporate alternative methods for the composition of services, such as an algorithm based on MoSeL [62] or different tools that are available in the Plan-jETI collection of planning algorithms.

## List of abbreviations

API: Application Programming Interface
BLAST: Basic Local Alignment Search Tool
BNF: Backus-Naur Form
BiBiServ: Bielefeld Bioinformatics Server
CTL: Computation Tree Logic
DDBJ: DNA Data Bank of Japan
DNA: DeoxyriboNucleic Acid
EBI: European Bioinformatics Institute
EMBOSS: European Molecular Biology Open Software Suite
GEAR: Game-based Easy And Reversed model checking tool
GO: Gene Ontology
GUI: Graphical User Interface
IT: Information Technology
(j)ABC: Application Building Center (Java implementation)
(j)ETI: Electronic Tool Integration platform (Java implementation)
LTL: Linear Time Logic
MoSeL: Monadic Second order Logic
OBO: Open Bioinformatics Ontologies
OWL: Web Ontology Language
RDF: Resource Description Framework RNA: RiboNucleic Acid
S3C: Semantic Service Selection Contest
SAWSDL: Semantic Annotations for WSDL
SIB: Service-Independent Building block
SLG: Service Logic Graph
SLTL: Semantic Linear Time Logic SSWAP: Simple Semantic Web Architecture and Protocol
SWS: Semantic Web Services
URI: Uniform Resource Identifier
W3C: World Wide Web Consortium
WSDL: Web Service Description Language.
